# An Inebriate Asylum

**Published:** 1884-12

**Authors:** 


### AN INEBRIATE ASYLUM.

Hon. R. L. Berner of Monroe county, has introduced a bill in the legislature providing for the establishment of an Inebriate Asylum by the State. This is a measure that has frequently, of late, been brought to the attention of the legislature by a committee of the State Medical Society, but heretofore has never met with any favor at their hands.

It is to be hoped that Mr. Berner will be able to get the bill through. There is no general law that the legislature can enact that will be of greater benefit than this.

We cannot see how any sane man can get his consent to vote against a measure of this kind. The unfortunate class that is meant to be benefited by this act are just as much entitled to protection and care from the State, as if they were insane from any other cause. The fact that it is the result of their own actions is no reason why the State should throw them off and refuse to care for them. We believe that there are hundreds who go on from bad to worse, and finally die, shunned and despised, for want of an institution of the kind provided by this bill. They lose all self-control and respect, if they are left alone to battle with the world, when they might, and frequently would be reclaimed and become good and useful citizens of the State if there was only some place of the kind to care for them. We feel satisfied that if the legislature will give this subject the study it deserves, they will pass this bill, and Georgia will no longer be pointed at as a State that makes no pretentions to care for inebriates. We would suggest that every medical society in the State memorialize the legislature to pass this bill.

